# Red blood cell transfusion thresholds in pediatric septic patients

**DOI:** 10.1186/cc9848

**Published:** 2011-03-11

**Authors:** O Karam, M Tucci, T Ducruet, H Hume, J Lacroix, F Gauvin

**Affiliations:** 1 Hopitaux Universitaires de Genève, Switzerland; 2 CHU Sainte-Justine, Montréal, Canada

## Introduction

In children with severe sepsis or septic shock, the optimal red blood cell (RBC) transfusion threshold is unknown. We analyzed the subgroup of patients with sepsis in the TRIPICU (Transfusion Requirements in Pediatric Intensive Care Units) study in order to determine the impact of a restrictive versus liberal transfusion strategy on clinical outcome.

## Methods

This study is a subgroup analysis of a prospective multicenter randomized controlled trial (TRIPICU). One hundred and thirty-seven stabilized critically ill children (mean systemic arterial pressure >2 SD below normal mean for age and cardiovascular support not increased for at least 2 hours before enrolment), with a hemoglobin ≤9.5 g/dl within 7 days after PICU admission, were randomized to receive RBC transfusion if their hemoglobin dropped below either 7.0 g/dl (restrictive group) or 9.5 g/dl (liberal group).

## Results

In the restrictive group (69 patients), 30 patients did not receive any RBC transfusion, whereas only one patient in the liberal group (68 patients) was never transfused (*P *< 0.01). No clinically significant differences were found for the occurrence of new or progressive multiple organ dysfunction syndrome (18.8% vs. 19.1%; *P *= 0.97), for PICU length of stay (*P *= 0.74) or PICU mortality (*P *= 0.44) in the restrictive versus liberal group.

## Conclusions

In this subgroup analysis of stable septic children, we found no evidence that a restrictive red-cell transfusion strategy, as compared with a liberal one, increased the rate of new or progressive MODS. On the other hand, a restrictive transfusion threshold significantly reduced exposure to blood products. Our data suggest that a hemoglobin level of 7 g/dl may be safe for stabilized septic children but further studies are required to support this recommendation.
